# In vivo characterization of chimeric PCV DNA clones containing heterogeneous capsid protein nuclear localization signals (NLS)

**DOI:** 10.1186/1743-422X-10-16

**Published:** 2013-01-07

**Authors:** Jiangbing Shuai, Xiaofeng Zhang, Wujian Chen, Ke Li, Shan Wu, Yongqiang He, Weihuan Fang

**Affiliations:** 1 Zhejiang Entry-Exit Inspection and Quarantine Bureau, 126 Fuchun Road, Hangzhou 310016, China; 2 Institute of Preventive Veterinary Medicine, Zhejiang University, 388 Yuhangtang Road, Hangzhou 310058, China; 3 Yiwu Entry-Exit Inspection and Quarantine Bureau, 299 Chengbei Road, Yiwu, 322000, China

**Keywords:** Porcine circovirus, Nuclear localization signal, Chimeric, Replication

## Abstract

**Background:**

PCV ORF2 capsid protein was predicted to contribute to the control of replication via an interaction between the Cap and Rep proteins in the nucleoplasm. We previously showed that the nuclear localization signal (NLS) on the capsid protein plays an accessory role in the replication of PCV in vitro. To further evaluate the in vivo characteristics of NLS-chimeric PCV DNA clones, BALB/C mice were inoculated intranasally and intraperitoneally with the DNA clones.

**Results:**

As expected, no gross lesions were detected during the study of the inoculated animals. The chimeric PCV12-, PCV1-NLS2- and PCV2-NLS1-inoculated animals had significantly fewer and less severe histopathological lesions in lymphoid tissues than the PCV2-inoculated animals (P < 0.05). PCV12 induced a specific antibody response against PCV2 ORF2 comparable to that induced by wild-type PCV2 but demonstrated a shorter period of viremia and much lower level of virus loads in sera than those in PCV2-inoculated mice. Remarkably, the PCV2-NLS1 and PCV1-NLS2 chimeras replicated in inoculated mice and induced specific antibody responses but failed to produce viral antigens in the lymph nodes or a detectable viremia.

**Conclusions:**

The chimeric PCV2-NLS1 and PCV1-NLS2 demonstrated a lower replication level as compared with wild type of PCV2 or PCV1 in vivo, suggesting that ORF2 NLSs played an accessory role in PCV replication. The chimeric PCV12 is a good candidate for vaccination against PCV2 infection.

## Background

Porcine circovirus type 2 (PCV2) is the primary causative agent of postweaning multisystemic wasting syndrome (PMWS), which was first described in 1991 in Saskatchewan, Canada [1,2]; PCV1 was identified in a porcine kidney cell line (PK-15) and was non-cytopathic in pigs [3]. The genomes of both PCV1 and PCV2 contain a conserved stem-loop structure and an identical essential core element at the origin of DNA replication and replicate via a rolling cycle replication mechanism [4-8]. Further study revealed that the *cis*- and *trans*-acting replication factors of PCV1 and PCV2 were functionally interchangeable, indicating that the replication strategy may not be the main factor determining the distinct propagation efficiencies and pathogenicities of PCV1 and PCV2 [8].

In *geminiviridae*, which use the same mode of replication as PCV, failed nuclear localization of the coat protein results in a drastic reduction in viral genomic ssDNA accumulation, indicating that the capsid protein mediates viral DNA transport and plays a role in controlling viral DNA copy number [9,10]. The PCV capsid protein is expressed late in the infection cycle and colocalizes to the nucleoplasm together with the replication protein, indica-ting that,in addition to its role in encapsidation, the PCV capsid protein may contribute to control of replication via the interaction between Cap and Rep in the nucleoplasm [11,12]. Nuclear translocation of the PCV1 and PCV2 ORF2 proteins was mediated by functional stretches of nuclear localization signals (NLS) [13,14]. In a previous study, we found that the NLS sequences of the PCV1 and PCV2 capsid proteins were functionally interchangeable with respect to nuclear import and viral propagation, and the ORF2 NLS plays an accessory role in PCV replication in vitro [15]. In the present study, the in vivo characteristics of chimeric PCV DNA clones containing heterogeneous capsid protein NLSs were studied.

## Results

### In vitro characterization of the PCV12 DNA clone

**Figure 1:**
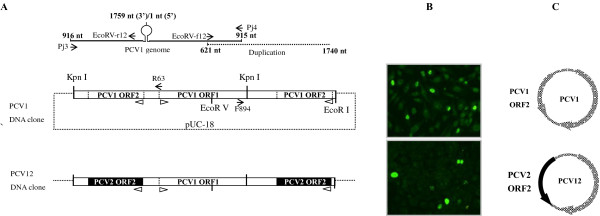
**(A) A schematic of the construction of the infectious PCV12 DNA clone. **(**B**) The PCV12 DNA clone was infectious when transfected in PK-15 cells. The cells were stained with a polyclonal antibody that recognizes the PCV2 capsid protein. (**C**) A schematic of the genome organization of the resulting chimeric PCV12 virus.

The PCV1, PCV2, PCV2-NLS1 and PCV1-NLS2 DNA clones were shown to be infectious in vitro in a previous study [15]. Detection of PCV2 capsid protein in approximately 10% of transfected cells (Figure 1B) using antibodies to the PCV2 ORF2 protein indicated that the PCV12 chimeric DNA clone could replicate in vitro and express the PCV2 capsid protein, as predicted. A 928-bp fragment that could only be the product of circular PCV12 genomes was amplified by primers R63 and F894 [15], while no large-sized product corresponding to the input recombinant plasmid was observed (data not shown).

**Figure 2:**
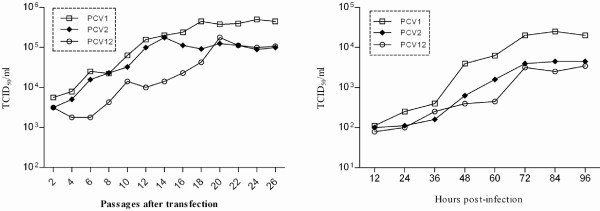
**In vitro viability (A) and one-step growth curve (B) of PCV12 produced by transfection of PK-15 cells with DNA clone. **The in vitro viability and growth characteristics of PCV1 and PCV2 were already evaluated in a previous study [15]_. _and were only used for comparison here. The infectious titers were determined by IFA according to the Reed-Muench method.

The initial titers of PCV1, PCV2 and PCV12 virus produced by transfected cells were all approximately 10^3.5^ TCID_50_/ml. The levels of all three viruses increased over the course of passaging and stabilized after approximately 20 passages at 10^5.6^, 10^5.0^ and 10^4.9^ TCID_50_/ml, respectively (Figure 2A. The lifecycle of PCV12 was examined further by one-step growth analysis. The infectious titers of PCV1, PCV2 and PCV12 were all approximately 10^2.0^ TCID_50_/ml after initial infection (Figure 2B) and increased gradually for all three viruses from 12 to 96 hours. PCV1 propagated most efficiently to reach a significantly higher titer of 10^4.3^ TCID_50_/ml by 96 h post-infection, whereas the infectious titers of PCV2 and PCV12 reached 10^3.6^ and 10^3.5^ TCID_50_/ml, respectively. This difference is most likely due to an adaptation of PCV1 that favors its growth in PK-15 cells because the PCV1 isolates used in these studies originated from PK-15 cell lines.

### Immunogenicity of the PCV1, PCV2, PCV12, PCV1-NLS2 and PCV2-NLS1 DNA clones in vivo

Prior to inoculation, twenty randomly selected mice were tested negatively for PCV1 and PCV2 antibodies. The cut-off values (mean ± 3 SD) were calculated using the OD values of serum samples from normal animals as a baseline.

Control animals in MEM-inoculated group were all negative for both PCV1 and PCV2 antibodies throughout the study (Table 1). In the group 2 and group 5 animals inoculated with PCV1 and PCV1-NLS2, seroconversion to PCV1 ORF2 first occurred at 14 dpi. The remaining mice in group 2 displayed antibodies to PCV1 by 28 dpi and remained positive until 42 dpi, while only six mice in group 5 seroconverted by the end of the study.

**Table 1:** Seroconversion to PCV1 or PCV2 antibodies in DNA-inoculated and control mice

**Group**	**Inoculum**^**a**^	**Antibodies tested**	**No. of sero-positive mice/no. tested**^**b **^**at dpi:**				
			**0**	**7**	**14**	**28**	**42**
1	MEM	PCV1 ORF2	0/4	0/4	0/4	0/4	0/4
		PCV2 ORF2	0/4	0/4	0/4	0/4	0/4
2	PCV1 DNA	PCV1 ORF2	0/4	0/4	2/4	4/4	4/4
3	PCV2 DNA	PCV2 ORF2	0/4	0/4	2/4	4/4	4/4
4	PCV12 DNA	PCV2 ORF2	0/4	0/4	2/4	3/4	4/4
5	PCV1-NLS2 DNA	PCV1 ORF2	0/4	0/4	1/4	2/4	3/4
6	PCV2-NLS1 DNA	PCV2 ORF2	0/4	0/4	2/4	3/4	3/4

^a^MEM was used as a negative control.

^b^Four mice were tested in each group at different time points following inoculation.

In the PCV2-inoculated group, seroconversion to PCV2 ORF2 antibodies was first detected in two of the four mice at 14 dpi. The remaining PCV2-inoculated mice displayed antibodies to to PCV2 by 28 dpi and remained positive until the end of the study at 42 dpi. In the group 4 mice inoculated with PCV12, seroconversion to PCV2 ORF2 specific antibodies first occurred at 14 dpi, and the rest of the mice seroconverted by 42 dpi. In mice inoculated with PCV2-NLS1, seroconversion to PCV2 antibodies was also first detected at 14 dpi and was found in three of the four mice by 28 dpi and 42 dpi.

**Figure 3:**
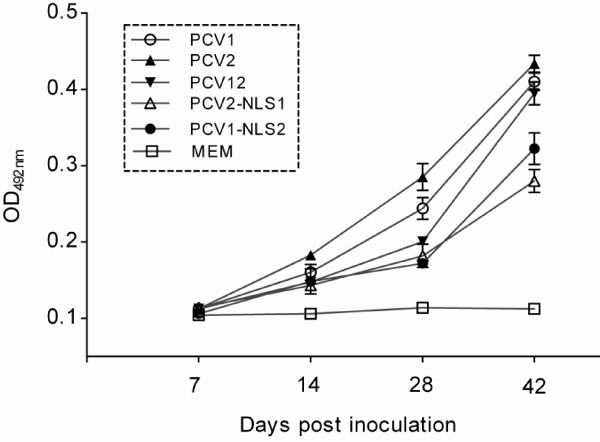
**PCV1- or PCV2-specific antibody dynamics in inoculated and control mice as determined by indirect ELISA. **Values at different dpi represent the mean OD_492 _of four mice per group; error bars represent the standard deviations.

The dynamics of PCV ORF2-specific antibody production in inoculated animals over the course of the experiment showed that all the DNA clones induced increasingly specific antibodies (Figure 3). By the end of the study at 42 dpi, the mean antibody levels in PCV1-, PCV2- and PCV12-inoculated mice were similar. However, the antibody titers in PCV1-NLS2- and PCV2-NLS1-inoculated mice were much lower than those induced by PCV1, PCV2 and PCV12.

### In vivo pathogenicity of the PCV1, PCV2, PCV12, PCV1-NLS2 and PCV2-NLS1 DNA clones

#### Clinical and pathological evaluation of inoculated mice

For all the inoculated animals, no clinical signs or gross lesions were observed throughout the experimental period. Sections of spleen, lung and mesenteric lymph nodes were evaluated microscopically, and the results are shown in Table 2. No microscopic lesions were observed in either the PCV1-inoculated group or the PCV1-NLS2-inoculated group mice at any time following inoculation. Only one mouse from the PCV2-inoculated group showed mild histiocytic broncho-interstitial pneumonia when examined at 28 dpi, and no lung lesions were observed in any mice from the other groups. Mild lymphoid depletion and histiocytic infiltration were detected in the spleens of four PCV2- and two PCV12-inoculated mice, as well as in one spleen sample from the PCV2-NLS1-inoculated group.

**Table 2:** Histopathological lesions in tissues of inoculated and control mice

**Group**	**Inoculum**^**a**^	**dpi**	**No. of mice with microscopic lesions/no. tested at dpi**^**b**^		
			**Lung**	**Lymph node**^**c**^	**Spleen**
**1**	MEM	7	0/4	0/4 (0.0)^I^	0/4 (0.0)^I^
		14	0/4	0/4 (0.0)^I^	0/4 (0.0)^I^
		28	0/4	0/4 (0.0)^I^	0/4 (0.0)^I^
		42	0/4	0/4 (0.0)^I^	0/4 (0.0)^I^
**2**	PCV1 DNA	7	0/4	0/4 (0.0)^I^	0/4 (0.0)^I^
		14	0/4	0/4 (0.0)^I^	0/4 (0.0)^I^
		28	0/4	0/4 (0.00)^I^	0/4 (0.0)^I^
		42	0/4	0/4 (0.00)^I^	0/4 (0.0)^I^
**3**	PCV2 DNA	7	0/4	2/4 (0.5)^I^	0/4 (0.0)^I^
		14	0/4	4/4 (1.0)^II^	1/4 (0.25)^I^
		28	1/4	4/4 (1.5)^II^	2/4 (0.75)^II^
		42	0/4	3/4 (1.75)^II^	1/4 (0.5)^II^
**4**	PCV12 DNA	7	0/4	0/4 (0.0)^I^	0/4 (0.0)^I^
		14	0/4	0/4 (0.0)^I^	0/4 (0.00)^I^
		28	0/4	2/4 (0.5)^I^	1/4 (0.25)^I^
		42	0/4	2/4 (0.5)^I^	1/4 (0.25)^I^
**5**	PCV1-NLS2 DNA	7	0/4	0/4 (0.0)^I^	0/4 (0.0)^I^
		14	0/4	0/4 (0.0)^I^	0/4 (0.0)^I^
		28	0/4	0/4 (0.0)^I^	0/4 (0.0)^I^
		42	0/4	0/4 (0.0)^I^	0/4 (0.0)^I^
**6**	PCV2-NLS1 DNA	7	0/4	0/4 (0.0)^I^	0/4 (0.0)^I^
		14	0/4	0/4 (0.0)^I^	0/4 (0.0)^I^
		28	0/4	1/4 (0.25)^I^	1/4 (0.25)^I^
		42	0/4	1/4 (0.25)^I^	0/4 (0.0)^I^

^a^MEM was used as a negative control.

^b^Four mice were tested in each group at different time points following inoculation.

^c^Values in parentheses are mean scores (0, normal; 1, mild; 2, moderate; 3, severe) for lymphoid depletion and histiocytic infiltration of 4 mice in each group. Different superscripts (^I ^and ^II^) indicate that the mean scores differ significantly between the groups (*P* < 0.05).

**Figure 4:**
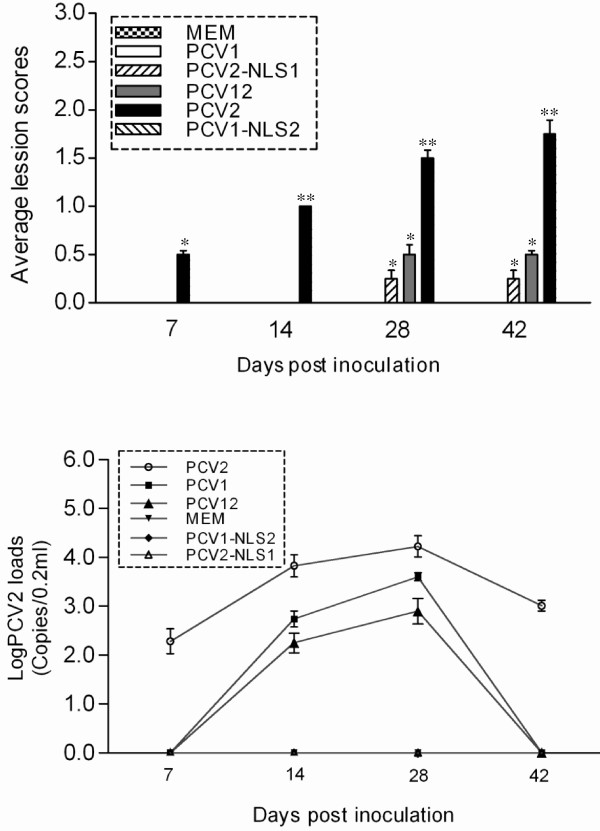
**(A) Evaluation of histopathological lesions within lymph nodes. **The values shown represent the mean scores of four mice per group. The average lesion values of mice in groups indicated by ** were significantly different from those of mice in groups indicated by *. (**B**) Quantification by real-time PCR of viremia in serum samples from inoculated mice. The values shown represent the mean DNA loads in the sera of four mice per group; error bars represent the standard deviations.

Compared with those in other tissues, lesions in lymph node from inoculated animals occurred with higher frequency and severity. Four PCV12- and two PCV2-NLS1-inoculated mice showed very mild lesions in the lymph nodes. However, mild-to-moderate lymph node lesions were detected in thirteen of the sixteen mice in the PCV2-inoculated group at 14, 28 and 42 dpi. The lesion scores for mice in the PCV12- and PCV2-NLS1-inoculated groups showed no significant differences versus those for mice in the PCV1-inoculated group (Table 2, Figure 4A) but were significantly different from those for mice in the PCV2-inoculated group (*P* < 0.05).

#### Evaluation of viremia by TaqMan PCR in sera of inoculated mice

Control animals in the MEM-inoculated group were all negative for PCV1- or PCV2-associated viremia throughout the study (Table 3). Viral DNA was not detected in the sera of mice inoculated with PCV1-NLS2 in group 5 or in the sera of mice inoculated with PCV2-NLS1 in group 6. In the group 3 mice inoculated with PCV2, viremia was first detected in two animals at 7 dpi and lasted until the end of the study, with a total of eleven out of twelve mice showing viremia. The group 2 and group 4 mice inoculated with PCV1 and PCV12 displayed shorter periods of viremia, the earliest-occurring instances of which were detected at 7 dpi in one mouse in each group and the latest-occurring instances of which were detected at 14 dpi in one and two mice per group, respectively. The copy number of PCV2 genomic DNA in PCV2-inoculated mice ranged from 10^2.3^ to 10^3.8^ copies/0.2 ml and peaked at 28 dpi (Figure 4B). Viral loads in PCV1- and PCV12-inoculated mice were 10^2.7^ and 10^2.3^ copies/0.2 ml, respectively, at 14 dpi and 10^3.6^ and 10^2.9^ copies/0.2 ml, respectively, at 28 dpi. Of further note, the viral loads in mice from the PCV1- and PCV12-inoculated groups were much lower than those in PCV2-inoculated mice (*P* < 0.05) over the course of the study.

**Table 3:** Detection of viremia in sera of inoculated and control mice by Taqman PCR

**Group**	**Inoculum**^**a**^	**No. of mice with viremia/no. tested at dpi**^**b**^**:**				
		**0**	**7**	**14**	**28**	**42**
1	MEM	0/4	0/4	0/4	0/4	0/4
2	PCV1 DNA	0/4	0/4	1/4	1/4	0/4
3	PCV2 DNA	0/4	2/4	4/4	4/4	3/4
4	PCV12 DNA	0/4	0/4	1/4	2/4	0/4
5	PCV1-NLS2 DNA	0/4	0/4	0/4	0/4	0/4
6	PCV2-NLS1 DNA	0/4	0/4	0/4	0/4	0/4

^a^MEM was used as negative control.

^b^Four mice were tested in each group at different time points following inoculation.

#### Detection of viral antigen in lymph nodes

Immunohistochemical detection of PCV ORF2-specific antigen was performed on lymph nodes from mice necropsied at 28 and 42 dpi. As shown in Table 4, lymph nodes from the MEM-, PCV1-, PCV1-NLS2- and PCV2-NLS1-inoculated mice were negative for both PCV1 ORF2 and PCV2 ORF2 antigen. In the PCV2-inoculated group, PCV2 ORF2 protein was detected in the lymph nodes of three out of the four animals collected at 28 dpi and four out of the four animals collected at 42 dpi. PCV2 ORF2 was also detected in the lymph nodes of only one out of the four mice collected at both 28 and 42 dpi from the chimeric PCV12-inoculated group. Furthermore, the mean scores representing the levels of PCV ORF2 antigen in the tissues of PCV12-inoculated animals were similar to those of the control and PCV1-inoculated groups but were significantly different from the mean scores of PCV2-inoculated mice at either 28 or 42 dpi (*P* < 0.05).

**Table 4:** Immunohistochemical detection of viral antigen in lymph nodes of inoculated mice

**Group**	**Inoculum**^**a**^	**No. of IHC positive mice /no. tested at dpi**^**b**^**:**	
		**28**	**42**
1	MEM	0/4(0.0)^I^	0/4(0.0)^I^
2	PCV1 DNA	0/4(0.0)^I^	0/4(0.0)^I^
3	PCV2 DNA	3/4(0.75)^II^	4/4(1.0)^II^
4	PCV12 DNA	1/4(0.25)^I^	1/4(0.25)^I^
5	PCV1-NLS2 DNA	0/4(0.0)^I^	0/4(0.0)^I^
6	PCV2-NLS1 DNA	0/4(0.0)^I^	0/4(0.0)^I^

^a^MEM was used as a negative control.

^b^Four mice were tested in each group at different time points following inoculation. Values in parentheses are mean levels of positive signal in lymph nodes. Different superscripts (^I ^and ^II^) indicate that the mean scores differ significantly between the groups (*P* < 0.05).

The viral genomic DNAs were amplified from lymph nodes of inoculated mice as described in the previous study [15]. Sequencing of the DNAs showed that all the viruses were genetically stable in vivo during the course of study, as no additional mutations or reversions were found in the genomes (data not shown).

## Discussion

Antibodies to PCV2 have been detected in various species other than pigs, including humans, mice and cattle [16-18]. The mechanism of PCV2-induced pathogenesis in mice is unknown. Quintana et al. reported that no microscopic lesions reminiscent of those observed with PCV2 infection in pigs were detected in inoculated mice, which might be explained by the low dose of the inoculum and the administration route [19]. However, several studies in which PCV2 was shown to be capable of replicating in mice and caused lesions similar to those observed in pigs have demonstrated that mice can indeed be used for an experimental model of PCV2 infection [20-22]. We showed previously that the NLS sequences of the viral capsid proteins were functionally interchangeable between PCV1 and PCV2 with respect to nuclear import and viral propagation. Those findings also revealed that PCV ORF2 NLSs were involved in viral replication in vitro. However, we found that the ORF2 NLS was not responsible for the differences in the in vitro growth characteristics of PCV1 and PCV2 [15]. In the present study, BALB/c mice were introduced as an animal model for evaluating the immunogenicity and pathogenicity of PCV2 and chimeric PCVs using both intranasal and intraperitoneal administration routes as described elsewhere [16,20].

In the present study, no gross lesions were observed in the inoculated animals at any point in the experimental course. On the microscopic level, the chimeric PCV12-, PCV1-NLS2- and PCV2-NLS1-inoculated animals showed statistically similar lesion scores to those of the nonpathogenic PCV1-inoculated mice but had significantly fewer (P < 0.05) and less severe histopathological lesions than the PCV2-inoculated animals. Scoring of the specific viral antigen levels detected in lymph nodes by IHC resulted in a mean score for PCV12-inoculated animals that was significantly lower (P < 0.05) than that for PCV2-inoculated mice, suggesting that there is less viral antigen present within the lymphoid tissues after PCV12 infection. However, IHC showed no detectable antigen in the lymph nodes of PCV2-NLS1- and PCV1-NLS2- inoculated animals. Measurement of viral DNA in the sera by real time PCR also revealed no detectable viremia in the PCV1-NLS2- or PCV2-NLS1-inoculated mice during the study. The lack of viral antigen in the lymph nodes, the absence of viremia and the reduced number and severity of microscopic lesions in PCV2-NLS1- and PCV1-NLS2-inoculated animals could be due to the lower replication levels of the two chimeric viruses in vivo. However, the absence of detectable chimeric PCV1-NLS2 and PCV2-NLS1 viremia in sera did not affect the rate of seroconversion, as all the inoculated groups produced serum antibodies against PCV capsid protein by the end of the study, as consistent with a previous finding [23]. In addition, PCV1- and PCV12-inoculated mice had shorter periods of viremia and lower viral loads in their sera than did the PCV2-inoculated mice.

## Conclusions

The availability of chimeric DNA clones enables us to further study the viral replication mechanism and the functional relationships between viral genes. The present study revealed that the chimeric PCV12 DNA clone induced a specific antibody response to PCV2 ORF2 comparable to that induced by PCV2 but had limited pathogenicity in vivo similar to nonpathogenic PCV1, suggesting that chimeric PCV12 could be a candidate for vaccination against PCV2 infection. Most remarkably, the PCV1-NLS2 and PCV2-NLS1 chimeras also replicated and induced specific antibody responses in vivo. However, the lack of viral antigen in lymph nodes and absence of detectable viremia revealed that PCV1-NLS2 and PCV2-NLS1 had lower replication levels in vivo compared with wild-type PCV2 or PCV1, suggesting that the PCV1 and PCV2 ORF2 NLSs were functionally interchangeable and played an accessory role in PCV replication.

## Methods

### Construction of the chimeric PCV12 DNA clone

The strategy used for PCV12 DNA clone construction was similar to that used for PCV1 DNA clone construction [15]. Briefly, PCV2 ORF2 was amplified with primer pair AflII-f and MspI-r (Table 5). The primers ClaI-f and AflII-r were then used to amplify the whole recombinant plasmid sequence of pUC-PCV1, including the pUC-18 vector and the PCV1 genome without its ORF2 sequence. The expected PCR products were ligated together to create recombinant plasmid pUC-PCV12, which contained the PCV2 capsid gene cloned into the backbone of the PCV1 genome. The PCV12 genome was excised and recircularized. The duplicated fragment was then amplified from the circularized PCV12 genome with primers EcoRV-f12 and EcoRI-r12 and subcloned into pUC-PCV12 to generate the chimeric PCV12 DNA clone pIS-PCV12 (Figure 1A).

**Table 5:** Primers used in the construction of the PCV12 DNA clone

**DNA clones**	**Primer**	**Primer Sequences (5’-3’)**^**a**^	**Primer location**	**Application**
	ClaI-f	CAATCGATAACGCCTCCTTGGATACGTCATC	1694-1724 nt	PCV1 ΔORF2
	AflII-r	TAACTTAAGAATAAAAACCATTACGATGTGATAAC	995-1029 nt	
PCV12	AflII-f	TTCCTTAAGGGTTAAGTGGGGGG	1029-1051 nt	PCV2 ORF2
DNA clone	MspI-r	CGTTACCGGAGAAGAAGACAC	1696-1716 nt	
	EcoRV-f12	GCGATATCATGGAGAAGAAGTTGTTGT	649-675 nt	Duplicate
	EcoRI-r12	CCGAATTCTCTTTCACTTTTATAGGATG	1721-1748 nt	

^a^The restriction enzyme sites are underlined.

### In vitro characterization of PCV12

To determine the infectivity and in vitro growth characteristics of the progeny PCV12 viruses, PK-15 cells grown to 80% confluency in 6-well plates were transfected with 20 μg of PCV1, PCV2 and PCV12 DNA clone as described previously [15]. The cells in each well were collected 5 days post-transfection and were then frozen and thawed three times. Subsequently, the cell lysates were used to inoculate fresh PK-15 cells growing in T-25 flasks, which were incubated for five days and then passaged serially 26 times. The in vitro viability and growth activity of the progeny viruses were evaluated by IFA as described previously [15].

### In vivo experimental design

One hundred and sixteen 7-week-old BALB/C mice were used for the in vivo experiment, twenty of which were randomly selected before inoculation to undergo nucleic acid and serological screening for PCV to confirm that the animals used in the study were initially PCV-free. The remaining mice were divided into six groups of sixteen animals each and housed in pens in separate rooms, where they were acclimatized for 7 days before inoculation. As a negative control, the mice in group 1 were inoculated intranasally and intraperitoneally with culture fluid (MEM). Mice in groups 2 and 3 were inoculated intranasally and intraperitoneally with 0.2 ml (50 μg) of the PCV1 and PCV2 DNA clones. Group 4 animals were inoculated with 0.2 ml (50 μg) of the PCV12 DNA clone. Animals in groups 5 and 6 were inoculated with 0.2 ml (50 μg) of the chimeric PCV1-NLS2 and PCV2-NLS1 DNA clones [15]. All mice were monitored daily for health status and possible clinical signs. Four mice in each group were euthanized by bleeding at 7, 14, 28 and 42 days post-inoculation (dpi). Blood and tissue samples were collected from euthanized mice and stored at −80°C for further studies. The animal experiments in this study were approved by the Animal Welfare Committee of Zhejiang University (protocol No. 20100134).

### Serological study

Antibodies to PCV1 or PCV2 in the serum of each euthanized mouse were detected by modified indirect ELISA based on the recombinant ORF2 capsid protein of PCV1 or PCV2 as described previously [24,25].

### Pathological analysis

For microscopic study, sections of spleen, lung and mesenteric lymph nodes were fixed in 4% phosphate-buffered paraformaldehyde. The sections were then dehydrated, embedded in paraffin, stained with hematoxylin and eosin (HE) and finally evaluated under a microscope. Lesion scores for lymphoid tissues were estimated in blinded samples based on the level of lymphoid depletion and histiocytic infiltration, with scores ranging from 0 (normal or no lymphoid depletion) to 3 (severe lymphoid depletion and histiocytic infiltration).

### Viral detection by taqman PCR

Blood samples from euthanized mice were collected during necropsy and were stored at −80°C before DNA extraction. Viral DNA was extracted from serum using the QIAamp DNA Blood kit (QIAGEN, USA) according to the manufacturer’s instructions. The amount of PCV DNA obtained from serum was determined using PCV1 or PCV2 ORF2-based primer pairs and probes [15].

### Immunohistochemistry (IHC) analysis

Specific antigens of PCV1, PCV2 and the three chimeric viruses were detected by IHC in paraffin-embedded sections of lymph node collected at 28 and 42 dpi using polyclonal antiserum to PCV1 or PCV2 according to the procedures described previously [23]. The polyclonal antiserum to PCV1 or PCV2, which was raised in rabbits by immunization with PCV1 and PCV2 viral proteins, have been confirmed as type-specific by immunofluorescence assay and western blot [25]. The amounts of specific antigen distributed in the tissues were scored blindly on a scale of 0 (for no signal) to 3 (for a strong positive signal).

### Statistical analysis

Results were presented as averages ± the standard deviations. Associations between rates of pathological lessions (normal, mild, moderate and severe) and amounts of viral antigens in the lymph nodes (negative, mild, moderate and strong) were assessed using the χ^2^ test. Differences were considered significant when P < 0.05.

## Competing interests

The authors declare that they have no competing interests.

## Authors’ contributions

JS designed the whole project, carried out the DNA clone construction, performed data analysis and drafted the manuscript. XZ contributed to the construction of PCV12DNA clone, serological study and histopathological analysis. WC performed in vitro characterization of PCV12 DNA clone. KL contributed to serological and pathological study. SW helped to conduct the IHC analysis. YH preformed real time PCR. WF supervised the project, participated in the design of the study and data interpretation, and helped to draft the manuscript. All authors have read and approved the final manuscript.
